# On Pell, Pell-Lucas, and balancing numbers

**DOI:** 10.1186/s13660-017-1599-1

**Published:** 2018-01-04

**Authors:** Gül Karadeniz Gözeri

**Affiliations:** Department of Mathematics, Faculty of Science, Istanbul University, Vezneciler, Istanbul, Turkey

**Keywords:** Pell numbers, Pell-Lucas numbers, balancing numbers, perfect squares, divisibility properties, Pell equations

## Abstract

In this paper, we derive some identities on Pell, Pell-Lucas, and balancing numbers and the relationships between them. We also deduce some formulas on the sums, divisibility properties, perfect squares, Pythagorean triples involving these numbers. Moreover, we obtain the set of positive integer solutions of some specific Pell equations in terms of the integer sequences mentioned in the text.

## Introduction and preliminaries

Let *p* and *q* be two integers such that $d=p^{2}-4q\neq 0$ (to exclude a degenerate case). We set two integer sequences $U_{n}$ and $V_{n}$ by

1 $$ U_{n}=U_{n}(p,q)=pU_{n-1}-qU_{n-2} \quad \mbox{and} \quad V_{n}=V_{n}(p,q)=pV_{n-1}-qV_{n-2} $$

for $n\geq 2$ with initial values $U_{0}=0$, $U_{1}=1$ and $V_{0}=2$, $V_{1}=p$. The characteristic equation of (1) is $x^{2}-px+q=0$, and hence its roots are

2 $$ \alpha =\frac{p+\sqrt{d}}{2} \quad \mbox{and} \quad \beta =\frac{p-\sqrt{d}}{2}. $$

Their Binet formulas are

3 $$ U_{n}=\frac{\alpha ^{n}-\beta ^{n}}{\alpha -\beta} \quad \mbox{and} \quad V_{n}=\alpha ^{n}+\beta ^{n} $$

for $n\geq 0$.

Note that, in (1), $U_{n}(1,-1)=F_{n}$, Fibonacci numbers (sequence A000045 in OEIS), $V_{n}(1,-1)=L_{n}$, Lucas numbers (sequence A000032 in OEIS), $U_{n}(2,-1) = P_{n}$, Pell numbers (sequence A000129 in OEIS), and $V_{n}(2,-1)=Q_{n}$, Pell-Lucas numbers (sequence A002203 in OEIS) (for further details, see [1–6]).

Balancing numbers have been defined in [7] and [8]. A positive integer *n* is called a balancing number if the Diophantine equation

4 $$ 1+2+\cdots +(n-1)=(n+1)+(n+2)+\cdots +(n+r) $$

holds for some positive integer *r*, which is called a cobalancing number. From (4) we have $\frac{(n-1)n}{2}=rn+\frac{r(r+1)}{2}$, and so

5 $$ r=\frac{-(2n+1)+\sqrt{8n^{2}+1}}{2} \quad \mbox{and} \quad n=\frac{2r+1+\sqrt{8r^{2}+8r+1}}{2}. $$

Let $B_{n}$ denote the *n*th balancing number, and let $b_{n}$ denote the *n*th cobalancing number. Then by (5) $B_{n}$ is a balancing number if and only if $8B_{n}^{2}+1$ is a perfect square, and $b_{n}$ is a cobalancing number if and only if $8b_{n}^{2}+8b_{n}+1$ is a perfect square. So $C_{n}=\sqrt{8B_{n}^{2}+1}$ and $c_{n}=\sqrt{8b_{n}^{2}+8b_{n}+1}$ are integers, called the *n*th Lucas-balancing and *n*th Lucas-cobalancing number, respectively. The Binet formulas for balancing numbers are $B_{n}=\frac{\alpha ^{2n}-\beta ^{2n}}{4\sqrt{2}}$, $b_{n}=\frac{\alpha ^{2n-1}-\beta ^{2n-1}}{4\sqrt{2}}-\frac{1}{2}$, $C_{n}=\frac{\alpha ^{2n}+\beta ^{2n}}{2}$, and $c_{n}=\frac{\alpha ^{2n-1}+\beta ^{2n-1}}{2}$, respectively, where $\alpha =1+\sqrt{2}$ and $\beta =1-\sqrt{2}$ (for further details, see [9–12]).

Later balancing numbers were generalized to the *t*-balancing numbers (see [13]) for an integer $t\geq 2$. A positive integer *n* is called a *t*-balancing number if

6 $$ 1+2+\cdots +n=(n+1+t)+(n+2+t)+\cdots +(n+r+t) $$

for some positive integer *r*, which is called a *t*-cobalancing number. From (6) we observe that

7 $$ \begin{gathered}r = \frac{-(2n+2t+1)+\sqrt{8n^{2}+8n(1+t)+(2t+1)^{2}}}{2}, \\ n = \frac{(2r-1)+\sqrt{8r^{2}+8tr+1}}{2}. \end{gathered} $$

Let $B_{n}^{t}$ denote the *n*th *t*-balancing number, and let $b_{n}^{t}$ denote the *n*th *t*-cobalancing number. Then from (7) we see that $B_{n}^{t}$ is a *t*-balancing number if and only if $8(B_{n}^{t})^{2}+8B_{n}^{t}(1+t)+(2t+1)^{2}$ is a perfect square and that $b_{n}^{t}$ is a *t*-cobalancing number if and only if $8(b_{n}^{t})^{2}+8tb_{n}^{t}+1$ is a perfect square. So

8 $$ C_{n}^{t}=\sqrt{8\bigl(B_{n}^{t} \bigr)^{2}+8B_{n}^{t}(1+t)+(2t+1)^{2}} \quad\text{and}\quad c_{n}^{t}=\sqrt{8 \bigl(b_{n}^{t}\bigr)^{2}+8tb_{n}^{t}+1} $$

are integers, which are called the *n*th Lucas *t*-balancing and the *n*th Lucas *t*-cobalancing number (for further details, see [14]).

Santana and Diaz-Barrero [15], setting a sequence in Lemma 2 as

9 $$ a_{n}=P_{2n}+P_{2n+1}, $$

proved that it was a generalized Fibonacci sequence given by $a_{n+1}=6a_{n} -a_{n-1}$ for $n\geq 1$, with initial values $a_{0}=1$ and $a_{1}=7$. Since $P_{n}=\frac{\alpha ^{n}-\beta ^{n}}{\alpha -\beta} $ for $\alpha =1+\sqrt{2} $ and $\beta =1-\sqrt{2}$, we get

10 $$ a_{n}=\frac{\alpha ^{2n+1}+\beta ^{2n+1}}{2} $$

for $n\geq 0$. We can easily see that the sum of the first *n* nonzero terms of $a_{n}$ is

11 $$ \sum_{i=1}^{n}a_{i}= \frac{5a_{n}-a_{n-1}-6}{4}. $$

It is proved in [15, Thm. 1] that the sum of the first $4n+1$ nonzero terms of Pell numbers is

$$\begin{aligned} \sum_{i=1}^{4n+1}P_{i}= \biggl( \frac{\alpha ^{2n+1}+\beta ^{2n+1}}{2} \biggr) ^{2}. \end{aligned}$$

We conclude that the sum of the first $4n+1$ nonzero terms of Pell numbers is

$$\begin{aligned} \sum_{i=1}^{4n+1}P_{i}=a_{n}^{2}. \end{aligned}$$

Moreover, Santana and Diaz-Barrero [15] proved that the sum of the first $4n+1$ nonzero terms of Pell numbers is a perfect square:

$$\begin{aligned} \sum_{i=1}^{4n+1}P_{i}=\left ( \sum_{r=0}^{n} \left ( \begin{matrix} 2n+1 \\ 2r \end{matrix} \right ) 2^{r} \right ) ^{2}. \end{aligned}$$

Later, Tekcan and Tayat [16] proved that the sum of the first $2n+1$ nonzero terms of Pell numbers is a perfect square if *n* is even or half of a perfect square if *n* is odd. They proved that the sum of the first $2n+1$ nonzero terms of Pell numbers is

12 $$ \sum_{i=1}^{2n+1}P_{i}= \textstyle\begin{cases} ( \frac{\alpha ^{n+1}+\beta ^{n+1}}{2} ) ^{2} & \mbox{for even }n\geq 2, \\ \frac{ ( \frac{\alpha ^{n+1}-\beta ^{n+1}}{\sqrt{2}} ) ^{2}}{2} & \mbox{for odd }n\geq 1,\end{cases} $$

where $\alpha =1+\sqrt{2}$ and $\beta =1-\sqrt{2}$. Considering (12) and setting two integer sequences

13 $$ X_{n}=\frac{\alpha ^{n+1}+\beta ^{n+1}}{2}\quad \mbox{and}\quad Y_{n}= \frac{\alpha ^{n+1}-\beta ^{n+1}}{\sqrt{2}} $$

for $n\geq 0$, they proved that the sum of the first $4n+1$ nonzero terms of Pell numbers is

$$\begin{aligned} \sum_{i=1}^{4n+1}P_{i}= \bigl[2X_{n}^{2}-2X_{n}Y_{n-1}+(-1)^{n+1} \bigr]^{2} \end{aligned}$$

for $n\geq 1$.

In this paper, we give several results on the sequences $a_{n}$, $X_{n}$, $Y_{n}$, $B_{n}$, $b_{n}$, $Q_{n}$, including sums, divisibility properties, perfect squares, and integer solutions of some specific Pell equations.

## Results and discussion

In this section, we derive our main results.

### Sums and divisibility properties

In this subsection, we deal with the sums and divisibility properties of numbers mentioned. First, we reformulate (11) in terms of Pell and Pell-Lucas numbers as follows.

#### Theorem 1

*The sum of the first* *n* *nonzero terms of* $a_{n}$ *is*

$$\begin{aligned} \sum_{i=1}^{n}a_{i}= \textstyle\begin{cases} \frac{17P_{2n-1}+7P_{2n-2}-3}{2}, \\ \frac{5Q_{2n}+2Q_{2n-1}-6}{4}. \end{cases}\displaystyle \end{aligned}$$

#### Proof

Since $a_{1}+a_{2}+\cdots +a_{n}=\frac{5a_{n}-a_{n-1}-6}{4}$ by (11) and since $a_{n}=P_{2n}+P_{2n+1}$ by (9), we deduce that

$$\begin{aligned} \sum_{i=1}^{n}a_{i} =& \frac{5a_{n}-a_{n-1}-6}{4} \\ =&\frac{5(P_{2n}+P_{2n+1})-(P_{2n-2}+P_{2n-1})-6}{4} \\ =&\frac{5P_{2n}+5(2P_{2n}+P_{2n-1})-P_{2n-2}-P_{2n-1}-6}{4} \\ =&\frac{15(2P_{2n-1}+P_{2n-2})+4P_{2n-1}-P_{2n-2}-6}{4} \\ =&\frac{17P_{2n-1}+7P_{2n-2}-3}{2}. \end{aligned}$$

The second result can be proved similarly. □

#### Theorem 2

*For the sequences* $a_{n}$, $X_{n}$, $Y_{n}$, $B_{n}$, $b_{n}$, $Q_{n}$, *and* $P_{n}$, *we have*:

1. $X_{n}=P_{n+1}+P_{n}$ *for* $n\geq 0$, *and the sum of the first* *n* *nonzero terms of* $X_{n}$ *is*
  $$\begin{aligned} \sum_{i=1}^{n}X_{i}=P_{n}+2P_{n+1}-2. \end{aligned}$$
  *Moreover*, $Y_{n}=P_{n+2}-P_{n}$ *for* $n\geq 0$, *and the sum of the first* *n* *nonzero terms of* $Y_{n}$ *is*
  $$\begin{aligned} \sum_{i=1}^{n}Y_{i}=P_{n}+3P_{n+1}-3. \end{aligned}$$
2. $a_{n}=X_{2n}$ *for* $n\geq 0$ *or* $a_{n}=X_{2n-1}+Y_{2n-1}$ *for* $n\geq 1$; $Q_{n+1}=2X_{n}$ *and* $Q_{n+2}-Q_{n+1}=2Y_{n}$ *for* $n\geq 0$; $a_{n}=\frac{Q_{2n+1}}{2}$ *and* $B_{n}=\frac{P_{n}Q_{n}}{2}$ *for* $n\geq 0$, *and* $b_{n}=\frac{P_{n}Q_{n-1}+P_{n-1}Q_{n}-2}{4}$ *for* $n\geq 1$.

#### Proof

(1) Let

14 $$ X_{n}=T_{1}\alpha ^{n}+T_{2}\beta ^{n} $$

for some $T_{1}$ and $T_{2}$. If we take $n=0$ and $n=1$, then we have the system of equations $T_{1}+T_{2}=1$ and $T_{1}\alpha +T_{2}\beta =3 $. This system of equations has the solution $T_{1}=\frac{\alpha +1}{\alpha -\beta} $ and $T_{2}=\frac{-\beta -1}{\alpha -\beta} $. So (14) becomes

$$\begin{aligned} X_{n}& =T_{1}\alpha ^{n}+T_{2}\beta ^{n} \\ & = \biggl( \frac{\alpha +1}{\alpha -\beta} \biggr) \alpha ^{n}+ \biggl( \frac{-\beta -1}{\alpha -\beta} \biggr) \beta ^{n} \\ & =\frac{(\alpha +1)\alpha ^{n}-(\beta +1)\beta ^{n}}{\alpha -\beta} \\ & =\frac{\alpha ^{n+1}-\beta ^{n+1}}{\alpha -\beta} +\frac{\alpha ^{n}-\beta ^{n}}{\alpha -\beta} \\ & =P_{n+1}+P_{n}, \end{aligned}$$

as we wanted. Since $X_{n}=P_{n}+P_{n+1}$ and $P_{1}+P_{2}+\cdots +P_{n}=\frac{P_{n}+P_{n+1}-1}{2}$, we easily deduce that

$$\begin{aligned} \sum_{i=1}^{n}X_{i}=\sum _{i=1}^{n}(P_{i}+P_{i+1})=P_{n}+2P_{n+1}-2. \end{aligned}$$

Similarly, it can be shown that $Y_{n}=P_{n+2}-P_{n}$ for $n\geq 0$ and $Y_{1}+Y_{2}+\cdots +Y_{n}=P_{n}+3P_{n+1}-3$.

(2) Since $a_{n}=\frac{\alpha ^{2n+1}+\beta ^{2n+1}}{2}$ and $X_{n}=\frac{\alpha ^{n+1}+\beta ^{n+1}}{2}$, we get

$$\begin{aligned} X_{2n}=\frac{\alpha ^{2n+1}+\beta ^{2n+1}}{2}=a_{n}. \end{aligned}$$

Similarly, since $X_{n}=P_{n+1}+P_{n}$ and $Y_{n}=P_{n+2}-P_{n}$, we get $X_{n}+Y_{n}=P_{n+1}+P_{n+2}$, and hence

$$\begin{aligned} X_{2n-1}+Y_{2n-1}=P_{2n}+P_{2n+1}=a_{n}. \end{aligned}$$

The remaining cases can be proved similarly. □

#### Theorem 3

*Let* $P_{n}$ *denote the* *nth Pell number*.

1. *If* $n\geq 2$ *is even*, *then*
  $$\begin{aligned} 1+\sum_{i=1}^{2n-1}{P_{i}} =& \textstyle\begin{cases} (\frac{Q_{n}}{2})^{2}, \\ (2B_{\frac{n}{2}}+2b_{\frac{n}{2}}+1)^{2},\end{cases}\displaystyle \end{aligned}$$
  *and if* $n\geq 1$ *is odd*, *then*
  $$\begin{aligned} \sum_{i=1}^{2n-1}{P_{i}} =& \textstyle\begin{cases} (\frac{Q_{n}}{2})^{2}, \\ (b_{\frac{n+3}{2}}-B_{\frac{n+1}{2}}-B_{\frac{n-1}{2}}-b_{\frac{n-1}{2}})^{2}.\end{cases}\displaystyle \end{aligned}$$
2. *If* $n\geq 2$ *is even*, *then*
  $$\begin{aligned} \sum_{i=1}^{4n+1}{P_{i}}= \bigl(2P_{n+1}^{2}-2P_{n}^{2}-1 \bigr)^{2}, \end{aligned}$$
  *and if* $n\geq 1$ *is odd*, *then*
  $$\begin{aligned} \sum_{i=1}^{4n+1}{P_{i}}= \bigl(2P_{n+1}^{2}-2P_{n}^{2}+1 \bigr)^{2}. \end{aligned}$$

#### Proof

(1) Let *n* be even, say $n=2k$ for some positive integer *k*. Since $P_{1}+P_{2}+\cdots +P_{n}=\frac{P_{n}+P_{n+1}-1}{2}$, we easily get

$$\begin{aligned} 1+\sum_{i=1}^{2n-1}{P_{i}} =&1+ \frac{P_{2n-1}+P_{2n}-1}{2} \\ =&\frac{P_{2n-1}+P_{2n}+1}{2} \\ =&\frac{\frac{\alpha ^{4k-1}-\beta ^{4k-1}+\alpha ^{4k}-\beta ^{4k}}{2\sqrt{2}}+1}{2} \\ =&\frac{\alpha ^{4k}(\alpha ^{-1}+1)+\beta ^{4k}(-\beta ^{-1}-1)+2\sqrt{2}}{4\sqrt{2}} \\ =&\frac{\alpha ^{4k}+\beta ^{4k}+2(\alpha \beta )^{2k}}{4} \\ =& \biggl( \frac{\alpha ^{2k}+\beta ^{2k}}{2} \biggr) ^{2} \\ =&\biggl(\frac{Q_{n}}{2}\biggr)^{2}. \end{aligned}$$

The other cases can be proved similarly. □

#### Theorem 4

*For the sequences mentioned*, *we have*

$$\begin{aligned}& \sum_{i=1}^{2n+1}{a_{i}}={a_{n}} {a_{n+1}},\qquad \sum_{i=1}^{2n}{a_{i}}=4B_{n+1}P_{2n} , \\& \sum_{i=1}^{2n+1}{B_{i}}={a_{n}} {B_{n+1}},\qquad \sum_{i=0}^{2n}{Y_{2i+1}}=2a_{n}P_{2n+2}, \\& \sum_{i=1}^{2n+1}{Q_{i}}={2(P_{2n+2}-1)}, \qquad \sum_{i=1}^{2n+1}{P_{i}}= \textstyle\begin{cases} (a_{\frac{n}{2}})^{2} , & n \textit{ even}, \\ Y_{n}P_{n+1}, & n \textit{ odd}, \end{cases}\displaystyle \\& \sum_{i=1}^{2n}{P_{2i}}={a_{n}} {P_{2n}}, \qquad \sum_{i=1}^{2n}{P_{i}}= \textstyle\begin{cases} 2P_{n}P_{n+1} , & n \textit{ even}, \\ a_{\frac{n-1}{2}}X_{n}, & n \textit{ odd}, \end{cases}\displaystyle \\& \sum_{i=1}^{2n}{X_{2i}}={8X_{n}} {P_{n+1}} {B_{n}}, \qquad \sum_{i=1}^{2n}{B_{i}}=a_{n}B_{n}, \\& \sum_{i=1}^{2n}{Q_{i}}={4b_{n+1}}, \qquad \sum_{i=1}^{2n}{B_{2i}}=P_{2n}P_{2n+1}X_{2n}C_{n}, \\& \sum_{i=1}^{2n}{Y_{2i-1}}={2a_{n}} {P_{2n}}, \qquad \sum_{i=1}^{2n}{a_{2i-1}}=a_{2n}P_{2n}X_{2n-1}, \\& \sum_{i=0}^{2n}{P_{2i+1}}={a_{n}} {P_{2n+1}}, \qquad \sum_{i=1}^{2n}{X_{2i-1}}=P_{2n+1}Y_{2n-1}, \\& \sum_{i=1}^{2n}{P_{2i-1}}={X_{2n-1}} {P_{2n}}, \qquad \sum_{i=0}^{2n}{a_{2i+1}}=a_{n}P_{2n+1}X_{4n+2}, \\& \sum_{i=1}^{2n}{a_{2i}}={B_{2n}} {a_{2n+1}}, \qquad \sum_{i=1}^{2n}{Q_{2i}}=2P_{2n+1}Y_{2n-1}. \end{aligned}$$

#### Proof

Since $\sum_{i=1}^{2n+1}{a_{i}}=\frac{ 29a_{2n}-5a_{2n-1} - 6}{ 4} $ by (11), we get

$$\begin{aligned} \sum_{i=1}^{2n+1}a_{i} =& \frac{29 ( \frac{\alpha ^{4n+1}+\beta ^{4n+1}}{2 } ) -5 ( \frac{\alpha ^{4n-1}+\beta ^{4n-1}}{2} ) -6}{4} \\ =&\frac{\alpha ^{4n}(29\alpha -5\alpha ^{-1})+\beta ^{4n}(29\beta -5\beta ^{-1})-12}{8} \\ =&\frac{\alpha ^{4n}(17+12\sqrt{2})+\beta ^{4n}(17-12\sqrt{2})-6}{4} \\ =&\frac{\alpha ^{4n+4}+\beta ^{4n+4}-6}{4} \\ =&\frac{\alpha ^{4n+4}+\beta ^{4n+4}-(\alpha \beta )^{2n+1}(\beta ^{2}+\alpha ^{2})}{4} \\ =& \biggl( \frac{\alpha ^{2n+1}+\beta ^{2n+1}}{2} \biggr) \biggl( \frac{\alpha ^{2n+3}+\beta ^{2n+3}}{2} \biggr) \\ =&a_{n}a_{n+1}. \end{aligned}$$

The other cases can be proved similarly. □

From Theorem 4 we have the following result.

#### Theorem 5

*For the divisibility properties*, *we have*

1. *If* *n* *is even*, *then* $a_{\frac{n}{2}}\vert \sum_{i=1}^{2n+1}{P_{i}}$, $P_{n}\vert \sum_{i=1}^{2n}{P_{i}}$, *and* $P_{n+1}\vert \sum_{i=1}^{2n}{P_{i}} $, *and if* *n* *is odd*, *then* $Y_{n}\vert \sum_{i=1}^{2n+1}{P_{i}} $, $P_{n+1}\vert \sum_{i=1}^{2n+1}{P_{i}} $, $a_{\frac{n-1}{2}}\vert \sum_{i=1}^{2n}{P_{i}} $, *and* $X_{n}\vert \sum_{i=1}^{2n}{P_{i}} $.
2. *For every* $n\geq 1$,
  $$\begin{aligned}& a_{n}\Bigg\vert \sum_{i=0}^{2n}{a_{2i+1}}, \qquad P_{2n+1}\Bigg\vert \sum_{i=0}^{2n}{a_{2i+1}}, \qquad X_{4n+2}\Bigg\vert \sum_{i=0}^{2n}{a_{2i+1}}, \qquad a_{2n}\Bigg\vert \sum_{i=1}^{2n}{a_{2i-1}}, \\& a_{n}\Bigg\vert \sum_{i=1}^{2n+1}{a_{i}}, \qquad a_{n+1}\Bigg\vert \sum_{i=1}^{2n+1}{a_{i}}, \qquad a_{n}\Bigg\vert \sum_{i=1}^{2n+1}{B_{i}}, \qquad B_{n+1}\Bigg\vert \sum_{i=1}^{2n+1}{B_{i}}, \\& a_{n}\Bigg\vert \sum_{i=1}^{2n}{B_{i}}, \qquad B_{n+1}\Bigg\vert \sum_{i=1}^{2n}{a_{i}}, \qquad P_{2n}\Bigg\vert \sum_{i=1}^{2n}{a_{i}}, \qquad B_{n}\Bigg\vert \sum_{i=1}^{2n}{B_{i}}, \\& a_{2n+1}\Bigg\vert \sum_{i=1}^{2n}{a_{2i}}, \qquad b_{n+1}\Bigg\vert \sum_{i=1}^{2n}{Q_{i}}, \qquad a_{n}\Bigg\vert \sum_{i=0}^{2n}{X_{2i+1}}, \qquad P_{2n}\Bigg\vert \sum_{i=1}^{2n}{a_{2i-1}}, \\& X_{2n-1}\Bigg\vert \sum_{i=1}^{2n}{a_{2i-1}}, \qquad a_{n}\Bigg\vert \sum_{i=1}^{2n}{P_{2i}}, \qquad P_{2n}\Bigg\vert \sum_{i=1}^{2n}{P_{2i}}, \qquad B_{2n}\Bigg\vert \sum_{i=1}^{2n}{a_{2i}}, \\& P_{2n}\Bigg\vert \sum_{i=1}^{2n}{Q_{2i-1}}, \qquad a_{n}\Bigg\vert \sum_{i=0}^{2n}{Q_{2i+1}}, \qquad P_{2n+1}\Bigg\vert \sum_{i=1}^{2n}{X_{2i-1}}, \qquad P_{n}\Bigg\vert \sum_{i=1}^{2n}{B_{2i-1}}, \\& Y_{2n-1}\Bigg\vert \sum_{i=1}^{2n}{X_{2i-1}}, \qquad a_{n}\Bigg\vert \sum_{i=0}^{2n}{Y_{2i+1}}, \qquad P_{2n+2}\Bigg\vert \sum_{i=0}^{2n}{Y_{2i+1}}, \qquad a_{n}\Bigg\vert \sum_{i=1}^{2n}{Y_{2i-1}}, \\& P_{2n}\Bigg\vert \sum_{i=1}^{2n}{Y_{2i-1}}, \qquad P_{2n+1}\Bigg\vert \sum_{i=0}^{2n}{B_{2i+1}}, \qquad a_{n}\Bigg\vert \sum_{i=0}^{2n}{B_{2i+1}}, \qquad X_{n-1}\Bigg\vert \sum_{i=1}^{2n}{B_{2i-1}}, \\& P_{2n+1}\Bigg\vert \sum_{i=1}^{2n}{B_{2i}}, \qquad P_{2n+1}\Bigg\vert \sum_{i=1}^{2n}{Q_{2i}}, \qquad Y_{2n-1}\Bigg\vert \sum_{i=1}^{2n}{Q_{2i}}, \qquad P_{2n}\Bigg\vert \sum_{i=1}^{2n}{B_{2i}}, \\& X_{n}\Bigg\vert \sum_{i=1}^{2n}{X_{2i}}, \qquad P_{n+1}\Bigg\vert \sum_{i=1}^{2n}{X_{2i}}, \qquad B_{n}\Bigg\vert \sum_{i=1}^{2n}{X_{2i}}, \qquad X_{2n}\Bigg\vert \sum_{i=1}^{2n}{B_{2i}}. \end{aligned}$$

Finally, we give the following result.

#### Theorem 6

*For the sequences* $a_{n}$, $X_{n}$, $Y_{n}$, $B_{n}$, $Q_{n}$, *and* $P_{n}$, *we have*

$$\begin{aligned}& \frac{ \sum_{i=0}^{2n}{a_{2i+1}} }{ \sum_{i=0}^{2n}{P_{2i+1}} }=X_{4n+2}, \qquad \frac{ \sum_{i=1}^{2n}{a_{2i-1}} }{ \sum_{i=1}^{2n}{P_{2i-1}} }=a_{2n}, \qquad \frac{ \sum_{i=0}^{2n}{B_{2i+1}} }{ \sum_{i=0}^{2n}{Q_{2i+1}} }=\frac{P_{2n+1}^{2}}{2}, \\& \frac{ \sum_{i=1}^{2n}{Q_{2i}} }{ \sum_{i=1}^{2n}{X_{2i-1}} }=2, \qquad \frac{ \sum_{i=1}^{2n}{Y_{2i-1}}}{ \sum_{i=1}^{2n}{P_{2i}} }=2. \end{aligned}$$

### Perfect squares

We see in (5) that $B_{n}$ is a balancing number if and only if $8B_{n}^{2}+1$ is a perfect square and that $b_{n}$ is a cobalancing number if and only if $8b_{n}^{2}+8b_{n}+1$ is a perfect square. Similarly, we can give the following result.

#### Theorem 7

*For every* $n\geq 1$,

1. $\frac{Q_{4n+2}-2}{4}$ *is a perfect square and* $\sqrt{\frac{ Q_{4n+2}-2}{4}}=a_{n}$;
2. $2P_{2n-1}^{2}-1$ *is a perfect square*, *and* $\sqrt{2P_{2n-1}^{2}-1} =a_{n-1}$;
3. $2P_{2n}^{2}+1$ *is a perfect square*, *and* $\sqrt{2P_{2n}^{2}+1} =C_{n}$;
4. $P_{2n+1}^{2}+P_{2n}P_{2n+2}$ *is a perfect square*, *and* $\sqrt{ P_{2n+1}^{2}+P_{2n}P_{2n+2}}=X_{2n}$;
5. $P_{2n}^{2}+P_{2n-1}^{2}+P_{4n}-1$ *is a perfect square*, *and* $\sqrt{ P_{2n}^{2}+P_{2n-1}^{2}+P_{4n}-1}=Y_{2n-1}$.

#### Proof

(1) Applying the Binet formulas, we deduce that

$$\begin{aligned} \sqrt{\frac{Q_{4n+2}-2}{4}} =&\sqrt{\frac{\alpha ^{4n+2}+\beta ^{4n+2}-2}{4}} =\sqrt{ \frac{\alpha ^{4n+2}+\beta ^{4n+2}+2(\alpha \beta )^{2n+1}}{4}} \\ =&\sqrt{ \biggl( \frac{\alpha ^{2n+1}+\beta ^{2n+1}}{2} \biggr) ^{2}}= \frac{ \alpha ^{2n+1}+\beta ^{2n+1}}{2}=a_{n}, \end{aligned}$$

as claimed. The other cases can be proved similarly. □

As in Theorem 7, we can give the following result.

#### Theorem 8

*For the sequences* $a_{n}$, $X_{n}$, $B_{n}$, $b_{n}$, $Q_{n}$, *and* $P_{n}$, *we have*

$$\begin{aligned}& \sqrt{\sum_{i=0}^{2n}{B_{2i+1}}}=a_{n}P_{2n+1}, \qquad \sqrt{\sum_{i=1}^{2n}{B_{2i-1}}}=2C_{n}X_{n-1}P_{n}, \\& \sqrt{\frac{ \sum_{i=0}^{2n}{Q_{2i+1}} }{2}}=a_{n},\qquad \sqrt{\sum _{i=1}^{2n}{Q_{2i-1}}}=2P_{2n}. \end{aligned}$$

#### Proof

Since $B_{n}=\frac{\alpha ^{2n}-\beta ^{2n}}{4\sqrt{2}}$, $P_{n}=\frac{\alpha ^{n}-\beta ^{n}}{2\sqrt{2}}$, and $a_{n}=\frac{\alpha ^{2n+1}+\beta^{2n+1}}{2}$, we get

$$\begin{aligned} \sqrt{\sum_{i=0}^{2n}{B_{2i+1}}} =&\sqrt{B_{1}+B_{3}+\cdots +B_{4n+1}} \\ =&\sqrt{\frac{\alpha ^{2}-\beta ^{2}}{4\sqrt{2}}+\frac{\alpha ^{6}-\beta ^{6} }{4\sqrt{2}}+\cdots +\frac{\alpha ^{8n+2}-\beta ^{8n+2}}{4\sqrt{2}}} \\ =&\sqrt{\frac{1}{4\sqrt{2}} \biggl( \frac{\alpha ^{8n+6}-\alpha ^{2}}{\alpha ^{4}-1}-\frac{\beta ^{8n+6}-\beta ^{2}}{\beta ^{4}-1} \biggr) } \\ =&\sqrt{\frac{\alpha ^{8n+4}-1}{32}+\frac{\beta ^{8n+4}-1}{32}} \\ =&\sqrt{ \biggl( \frac{\alpha ^{4n+2}+\beta ^{4n+2}-2(\alpha \beta )^{2n+1}}{8} \biggr) \biggl( \frac{\alpha ^{4n+2}+\beta ^{4n+2}+2(\alpha \beta )^{2n+1}}{4} \biggr) } \\ =&\sqrt{ \biggl( \frac{\alpha ^{2n+1}+\beta ^{2n+1}}{2} \biggr) ^{2} \biggl( \frac{\alpha ^{2n+1}-\beta ^{2n+1}}{2\sqrt{2}} \biggr) ^{2}} \\ =&a_{n}P_{2n+1}. \end{aligned}$$

The other cases can be proved similarly. □

### Continued fraction expansion

#### Theorem 9

*The continued fraction expansion of* $\frac{a_{n+1}}{a_{n}}$ *is*

$$\begin{aligned} \frac{a_{n+1}}{a_{n}}=\bigl[5;(1,4)_{n-1},1,6\bigr] \end{aligned}$$

*for* $n\geq 1$ (*here* $(x)_{k}$ *means that there are* *k* *successive terms ‘x’*).

#### Proof

Let $n=1$. Then

$$\begin{aligned} \frac{a_{2}}{a_{1}}=\frac{41}{7}=5+\frac{1}{1+\frac{1}{6}}=[5;1,6]. \end{aligned}$$

Let us assume that it is satisfied for $n-1$, that is, $\frac{a_{n}}{a_{n-1}} =[5;(1,4)_{n-2},1,6]$. Then we get

$$\begin{aligned} \frac{a_{n+1}}{a_{n}} =&\bigl[5;(1,4)_{n-1},1,6\bigr]=5+ \frac{1}{1+\frac{1}{4+\frac{1}{ \textstyle\begin{array}{c} 1+ \\ \cdots \\ 1+\frac{1}{6} \end{array}\displaystyle }}} \\ =&5+\frac{1}{1+\frac{1}{-1+5+\frac{1}{ \textstyle\begin{array}{c} 1+ \\ \cdots \\ 1+\frac{1}{6} \end{array}\displaystyle }}} =5+\frac{1}{1+\frac{1}{-1+\frac{a_{n}}{a_{n-1}}}} \\ =&5+\frac{1}{1+\frac{a_{n-1}}{a_{n}-a_{n-1}}}=5+\frac{a_{n}-a_{n-1}}{a_{n}}= \frac{6a_{n}-a_{n-1}}{a_{n}}. \end{aligned}$$

So it is true for all $n\geq 1$ since $a_{n+1}=6a_{n}-a_{n-1}$. □

### Companion matrix

The companion matrix for Pell numbers is $\big[ {\scriptsize\begin{matrix}{} 2 & 1 \cr 1 & 0\end{matrix}} \big] $. It is known that

15 $$ \left [ \begin{matrix} P_{n+1} & P_{n} \\ P_{n} & P_{n-1}\end{matrix} \right ] =\left [ \begin{matrix} 2 &1 \\ 1 &0\end{matrix} \right ] ^{n}. $$

So $P_{n+1}P_{n-1}-P_{n}^{2}=(-1)^{n}$, which known as the Cassini identity, is an immediate consequence of the matrix formula [17]. If we take the *n*th power of the matrix in the left side of (15), then we can give the following theorem, which can be proved by induction on *n*.

#### Theorem 10

*For the Pell numbers* $P_{n}$, *we have*

$$\begin{aligned} \left [ \begin{matrix} P_{n+1} & P_{n} \\ P_{n} & P_{n-1} \end{matrix} \right ] ^{n}= \left [ \begin{matrix} P_{n^{2}+1} & P_{n^{2}} \\ P_{n^{2}} & P_{n^{2}-1} \end{matrix} \right ] \end{aligned}$$

*for* $n\geq 1$.

### Pythagorean triples

It is known that the Pell numbers $P_{n}$ have a close connection with square triangular numbers, that is,

16 $$ \bigl( (P_{k-1}+P_{k})P_{k} \bigr) ^{2}=\frac{(P_{k-1}+P_{k})^{2} ( (P_{k-1}+P_{k})^{2}-(-1)^{k} ) }{2}. $$

Note that the left side of (16) describes a square number, whereas the right side describes a triangular number, so the result is a square triangular number (see [18]). Notice that if a right triangle has integer side lengths *a*, *b*, *c* (necessarily satisfying the Pythagorean theorem $a^{2}+b^{2}=c^{2}$), then $(a,b,c)$ is known as a Pythagorean triple. As Martin [19] described, Pell numbers can be used to form Pythagorean triples in which *a* and *b* are one unit apart, corresponding to right triangles that are nearly isosceles. For instance,

$$\begin{aligned} \bigl(2P_{n}P_{n+1},P_{n+1}^{2}-P_{n}^{2},P_{n+1}^{2}+P_{n}^{2} \bigr) \end{aligned}$$

is a Pythagorean triple. Now we can give the following theorem related to Pythagorean triples.

#### Theorem 11

$\sqrt{2}B_{2n},P_{2n-1}+P_{2n}$, *and* $B_{2n}+b_{2n}+1$ *form a Pythagorean triple*, *that is*,

$$\begin{aligned} (\sqrt{2}B_{2n})^{2}+(P_{2n-1}+P_{2n})^{2}=(B_{2n}+b_{2n}+1)^{2}, \end{aligned}$$

*and* $(P_{n}Q_{n}+2b_{n}+1)^{2}-8B_{n}^{2},\sqrt{2}P_{2n}$, *and* $X_{2n-1}$ *form a Pythagorean triple*, *that is*,

$$\begin{aligned} {}\bigl[ (P_{n}Q_{n}+2b_{n}+1)^{2}-8B_{n}^{2} \bigr]^{2}+(\sqrt{2} P_{2n})^{2}=X_{2n-1}^{2}. \end{aligned}$$

#### Proof

Applying the Binet formulas, we deduce that

$$\begin{aligned}& (\sqrt{2}B_{2n})^{2}+(P_{2n-1}+P_{2n})^{2} \\& \quad = \biggl[ \sqrt{2} \biggl( \frac{\alpha ^{4n}-\beta ^{4n}}{4\sqrt{2}} \biggr) \biggr] ^{2}+ \biggl( \frac{\alpha ^{2n-1}-\beta ^{2n-1}}{2\sqrt{2}}+\frac{ \alpha ^{2n}-\beta ^{2n}}{2\sqrt{2}} \biggr) ^{2} \\& \quad = \frac{\alpha ^{8n}-2(\alpha \beta )^{2n}+\beta ^{8n}}{16}+\frac{2\alpha ^{4n}+4(\alpha \beta )^{2n}+2\beta ^{4n}}{8} \\& \quad = \frac{(\alpha ^{4n}+\beta ^{4n})^{2}+4(\alpha ^{4n}+\beta ^{4n})+4}{16} \\& \quad = \biggl( \frac{\alpha ^{4n}(1+\alpha ^{-1})-\beta ^{4n}(1+\beta ^{-1})}{4 \sqrt{2}}+\frac{1}{2} \biggr) ^{2} \\& \quad = \biggl( \frac{\alpha ^{4n}-\beta ^{4n}}{4\sqrt{2}}+\frac{\alpha ^{4n-1}-\beta ^{4n-1}}{4\sqrt{2}}-\frac{1}{2}+1 \biggr) ^{2} \\& \quad = (B_{2n}+b_{2n}+1)^{2}. \end{aligned}$$

The other case can be proved similarly. □

### The Pell equation

Let *d* be any positive nonsquare integer, and let *N* be any fixed integer. Then the equation

17 $$ x^{2}-dy^{2}=\pm N $$

is known as a Pell-type equation; $x^{2}-dy^{2}=N$ is the positive Pell-type equation, and $x^{2}-dy^{2}=-N$ is the negative Pell-type equation. It is named after John Pell (1611-1685), a mathematician who searched for integer solutions to equations of this type in the seventeenth century. Ironically, Pell was not the first to work on this problem, nor did he contribute to our knowledge for solving it. Euler (1707-1783), who brought us the *ψ*-function, accidentally named the equation after Pell, and the name stuck.

For $N=1$, the Pell equation $x^{2}-dy^{2}=\pm 1$ is known as the classical Pell equation. The Pell equation $x^{2}-dy^{2}=1$ was first studied by Brahmagupta (598-670) and Bhaskara (1114-1185). Its complete theory was worked out by Lagrange (1736-1813), not Pell. It is often said that Euler (1707-1783) mistakenly attributed Brouncker’s (1620-1684) work on this equation to Pell. However, the equation appears in a book by Rahn (1622-1676), which was certainly written with Pell’s help: some say that it is entirely written by Pell. Perhaps Euler knew what he was doing in naming the equation. In 1657, Fermat stated (without giving proof) that the positive Pell equation $x^{2}-dy^{2}=1$ has an infinite number of solutions. If $(m,n) $ is a solution, that is, $m^{2}-dn^{2}=1$, then $(m^{2}+dn^{2},2mn)$ is also a solution since

$$\begin{aligned} \bigl(m^{2}+dn^{2}\bigr)^{2}-d(2mn)^{2}= \bigl(m^{2}-dn^{2}\bigr)^{2}=1. \end{aligned}$$

So the Pell equation $x^{2}-dy^{2}=1$ has infinitely many integer solutions. Later, in 1766, Lagrange proved that the Pell equation $x^{2}-dy^{2}=1$ has an infinite number of solutions if *d* is positive and nonsquare. The first nontrivial solution $(x_{1},y_{1})\neq (\pm 1,0)$ of this equation is called the fundamental solution from which all others are easily computed since $x_{n}+y_{n}\sqrt{d}=(x_{1}+y_{1}\sqrt{d})^{n}$ for $n\geq 1$ can be found using, for example, the cyclic method [20], known in India in the 12th century, or using the slightly less efficient but more regular English method [20] (17th century). There are other methods to compute this so-called fundamental solution, some of which are based on a continued fraction expansion of the square root of *d* given as follows. Let $\sqrt{d}=[m_{0};\overline{m_{1},m_{2},\ldots ,m_{l}}]$ denote the continued fraction expansion of period length *l*. Set $A_{-2}=0$, $A_{-1}=1$, $A_{k}=m_{k}A_{k-1}+A_{k-2}$ and $B_{-2}=1$, $B_{-1}=0$, $B_{k}=m_{k}B_{k-1}+B_{k-2} $ for nonnegative integers *k*. Then $C_{k}=\frac{A_{k}}{B_{k}}$ is the *k*th convergent of $\sqrt{d}$, and the fundamental solution of $x^{2}-dy^{2}=1$ is $(x_{1},y_{1})=(A_{l-1},B_{l-1})$ if *l* is even or $(A_{2l-1},B_{2l-1})$ if *l* is odd. Also, if *l* is odd, then the the fundamental solution of $x^{2}-dy^{2}=-1$ is $(x_{1},y_{1})=(A_{l-1},B_{l-1})$ (for further details on Pell equations, see [21–23]).

It is known that there is a connection between integer sequences and Pell equations. For instance, Olajas [9] gave the integer solutions to $x^{2}-5y^{2}=\pm 4$ as follows.

#### Theorem 12

([9, Thm. 2.17])

*The only solutions of the equation* $x^{2}-5y^{2}=\pm 4$ *are* $x=\pm L_{m}$ *and* $y=\pm F_{m}$, *where* $L_{m}$ *and* $F_{m}$ *are the* *mth terms of the Lucas and Fibonacci sequences*, *respectively*.

For integers *A* and *B* such that $A^{2}-4B\neq 0$ (to exclude a degenerate case), $R=\{R_{i}\}_{i=0}^{\infty }=R(A,B,R_{0},R_{1})$ is a second-order linear recurrence if the recurrence relation for $i\geq 2$

18 $$ R_{i}=AR_{i-1}-BR_{i-2} $$

holds for its terms and $R_{0},R_{1}$ are fixed integers. For the Pell equation $x^{2}-8y^{2}=1$, Liptai [24] proved the following:

#### Theorem 13

([24, Thm. 1])

*The terms of the second*-*order linear recurrence* $R(6,-1,1,6)$ *are the solutions of the equation* $z^{2}-8y^{2}=1$ *for some integer* *z*.

Now we can return to our main problem. We consider the integer solutions of the Pell equations

$$\begin{aligned} x^{2}-8y^{2}=1,\qquad x^{2}-2y^{2}=\pm 4,\quad \mbox{and}\quad x^{2}-8y^{2}=\pm 4. \end{aligned}$$

#### Theorem 14

1. *For the positive Pell equation* $x^{2}-8y^{2}=1$, *we have*:
  - *The integer solutions are* $(x_{n},y_{n})=(\frac{Q_{2n}}{2},\frac{P_{2n} }{2})$ *for* $n\geq 1$.
  - *The integer solutions satisfy the recurrence relation*
    $$\begin{aligned} x_{n}=\frac{5(Q_{2n-2}+Q_{2n-4})-Q_{2n-6}}{2}\quad \textit{and}\quad y_{n}= \frac{ 5(P_{2n-2}+P_{2n-4})-P_{2n-6}}{2} \end{aligned}$$
  *for* $n\geq 3$.
2. *The negative Pell equation* $x^{2}-8y^{2}=-1$ *has no integer solutions*.
3. *For the positive Pell equation* $x^{2}-2y^{2}=4$, *we have*:
  - *The integer solutions are* $(x_{n},y_{n})=(Q_{2n},Y_{2n-1})$ *for* $n\geq 1$.
  - *The integer solutions* $(x_{n},y_{n})$ *satisfy the recurrence relation*
    $$\begin{aligned} x_{n}=5(Q_{2n-2}+Q_{2n-4})-Q_{2n-6}\quad \textit{and}\quad y_{n}=5(Y_{2n-3}+Y_{2n-5})-Y_{2n-7} \end{aligned}$$
  *for* $n\geq 4$.
4. *For the negative Pell equation* $x^{2}-2y^{2}=-4$, *we have*:
  - *The integer solutions are* $(x_{n},y_{n})=(Q_{2n-1},Y_{2n-2})$ *for* $n\geq 1$.
  - *The integer solutions* $(x_{n},y_{n})$ *satisfy the recurrence relation*
    $$\begin{aligned} x_{n}=5(Q_{2n-3}+Q_{2n-5})-Q_{2n-7}\quad \textit{and}\quad y_{n}=5(Y_{2n-4}+Y_{2n-6})-Y_{2n-8} \end{aligned}$$
  *for* $n\geq 4$.
5. *For the positive Pell equation* $x^{2}-8y^{2}=4$, *we have*:
  - *The integer solutions are* $(x_{n},y_{n})=(Q_{2n},P_{2n})$ *for* $n\geq 1$ .
  - *The integer solutions* $(x_{n},y_{n})$ *satisfy the recurrence relation*
    $$\begin{aligned} x_{n}=5(Q_{2n-2}+Q_{2n-4})-Q_{2n-6}\quad \textit{and}\quad y_{n}=5(P_{2n-2}+P_{2n-4})-P_{2n-6} \end{aligned}$$
  *for* $n\geq 3$.
6. *For the negative Pell equation* $x^{2}-8y^{2}=-4$, *we have*:
  - *The integer solutions are* $(x_{n},y_{n})=(Q_{2n-1},P_{2n-1})$ *for* $n\geq 1$.
  - *The integer solutions* $(x_{n},y_{n})$ *satisfy the recurrence relation*
    $$\begin{aligned} x_{n}=5(Q_{2n-3}+Q_{2n-5})-Q_{2n-7}\quad \textit{and}\quad y_{n}=5(P_{2n-3}+P_{2n-5})-P_{2n-7} \end{aligned}$$
  *for* $n\geq 4$.

#### Proof

(1a) Notice that $Q_{n}=\alpha ^{n}+\beta ^{n}$ and $P_{n}=\frac{\alpha ^{n}-\beta ^{n}}{2\sqrt{2}}$. So

$$\begin{aligned} x^{2}-8y^{2}&=\biggl(\frac{Q_{2n}}{2} \biggr)^{2}-8\biggl(\frac{P_{2n}}{2}\biggr)^{2}= \frac{(\alpha ^{2n}+\beta ^{2n})^{2}-8 ( \frac{\alpha ^{2n}-\beta ^{2n}}{2\sqrt{2}} ) ^{2}}{4} \\ &=\frac{\alpha ^{4n}+2(\alpha \beta )^{2n}+\beta ^{4n}-(\alpha ^{4n}-2(\alpha \beta )^{2n}+\beta ^{4n})}{4} \\ &\begin{aligned} &=\frac{4(\alpha \beta )^{2n}}{4} \\ &=1 \end{aligned} \end{aligned}$$

since $\alpha \beta =-1$.

(1b)

$$\begin{aligned} \frac{5(Q_{2n-2}+Q_{2n-4})-Q_{2n-6}}{2} =&\frac{ 5Q_{2n-2}+5Q_{2n-4}-(2Q_{2n-5}+Q_{2n-6})+2Q_{2n-5}}{2} \\ =&\frac{5Q_{2n-2}+2(2Q_{2n-4}+Q_{2n-5})}{2} \\ =&\frac{2Q_{2n-1}+Q_{2n-2}}{2} \\ =&\frac{Q_{2n}}{2} \\ =&x_{n}. \end{aligned}$$

Similarly, it can be shown that $y_{n}=\frac{5(P_{2n-2}+P_{2n-4})-P_{2n-6}}{2} $.

(2) The negative Pell equation $x^{2}-8y^{2}=-1$ has no integer solutions since $\sqrt{8}=[2;\overline{1,4}]$, that is, the length of 2, which is even.

(3a) Notice that $Q_{n}=\alpha ^{n}+\beta ^{n}$ and $Y_{n}=\frac{\alpha ^{n+1}-\beta ^{n+1}}{\sqrt{2}}$. So

$$\begin{aligned} x^{2}-2y^{2} =&Q_{2n}^{2}-2Y_{2n-1}^{2}= \bigl(\alpha ^{2n}+\beta ^{2n}\bigr)^{2}-2 \biggl( \frac{\alpha ^{2n}-\beta ^{2n}}{\sqrt{2}} \biggr) ^{2} \\ =& \alpha ^{4n}+2(\alpha \beta )^{2n}+\beta ^{4n}- \bigl(\alpha ^{4n}-2(\alpha \beta )^{2n}+\beta ^{4n} \bigr) \\ =& 4(\alpha \beta )^{2n} \\ =& 4. \end{aligned}$$

(3b) Similarly, we get

$$\begin{aligned} 5(Q_{2n-2}+Q_{2n-4})-Q_{2n-6} =& 5Q_{2n-2}+5Q_{2n-4}-(2Q_{2n-5}+Q_{2n-6})+2Q_{2n-5} \\ =& 5Q_{2n-2}+2(2Q_{2n-4}+Q_{2n-5}) \\ =& 4Q_{2n-2}+Q_{2n-2}+2Q_{2n-3} \\ =& 2(2Q_{2n-2}+Q_{2n-3})+Q_{2n-2} \\ =& 2Q_{2n-1}+Q_{2n-2} \\ =& Q_{2n} \\ =& x_{n} \end{aligned}$$

The other cases can be proved similarly. □

It is known that there are a number of applications of Pell and Fibonacci sequences on the theory of numbers. For instance, Sellers proved in [25, Thm. 2.1] that the number of domino tilings of the graph $W_{4}\times P_{n-1}$ equals the product of the *n*th Fibonacci and Pell numbers for all $n\geq 2$. Also, there has been a connection between Diophantine quadruples and Fibonacci numbers. Recall that a set of *m* positive integers $\{a_{1},a_{2},\dots,a_{m}\}$ is called a Diophantine *m*-tuble if $a_{i}a_{j}+1 $ is a perfect square for all $1\leq i< j\leq m$ and is called a $D(n)$-*m*-tuble (or a Diophantine *m*-tuble with the property $D(n)$) if $a_{i}a_{j}+n$ is a perfect square for all $1\leq i< j\leq m$.

Cassini’s identity for Fibonacci number is $F_{n}F_{n+2}+(-1)^{n}=F_{n+1}^{2} $ and is the basis for the construction of so-called Hoggatt-Bergum’s quadruple. Hoggatt and Bergum [26] proved that, for any positive integer *k*, the set

$$\begin{aligned} \{F_{2k},F_{2k+2},F_{2k+4},4F_{2k+1}F_{2k+2}F_{2k+3} \} \end{aligned}$$

is a Diophantine quadruple. Later Morgado [27] and Horadam [28] generalized this result. The identity

$$\begin{aligned} F_{k-3}F_{k-2}F_{k-1}F_{k+1}F_{k+2}F_{k+3}+L_{k}^{2}= \bigl[F_{k}\bigl(2F_{k-1}F_{k+1}-F_{k}^{2} \bigr)\bigr]^{2} \end{aligned}$$

is known as the Morgado identity.

Using Fibonacci numbers, Dujella [29] defined the elliptic curve (see [30])

$$\begin{aligned} E_{k}:y^{2}=(F_{2k}x+1) (F_{2k+2}x+1) (F_{2k+4}x+1) \end{aligned}$$

and determined the integer points on it by terms of Fibonacci numbers when the rank of $E_{k}(\mathbb{Q})$ is 1.

It is known that there are several identities on Fibonacci and Lucas numbers. Some of them can be given as $4F_{k-1}F_{k+1}+F_{k}^{2}=L_{k}^{2}$ and $4F_{k-1}F_{k}^{2}F_{k+1}+1=(F_{k}^{2}+F_{k-1}F_{k+1})^{2}$. Using these, Dujella [31] obtained some quantities on $D(F_{k}^{2})$-quadruples

19 $$\begin{aligned} &\bigl\{ 2F_{k-1},2F_{k+1},2F_{k}^{3}F_{k+1}F_{k+2},2F_{k+1}F_{k+2}F_{k+3} \bigl(2F_{k+1}^{2}-F_{k}^{2}\bigr)\bigr\} , \\ &\bigl\{ F_{k-1},4F_{k+1},F_{k-2}F_{k-1}F_{k+1} \bigl(2F_{k}^{2}-F_{k-1}^{2} \bigr),F_{k}^{3}F_{k+2}F_{k+3}\bigr\} , \\ &\bigl\{ 4F_{k-1},F_{k+1},F_{k-2}F_{2k-2}F_{2k-1},F_{k}^{3}L_{k}L_{k+1} \bigr\} . \end{aligned}$$

As in (19), the set

$$\begin{aligned} \bigl\{ F_{k-3}F_{k-2}F_{k+1},F_{k-1}F_{k+2}F_{k+3},F_{k}L_{k}^{2},4F_{k-1}^{2}F_{k}F_{k+1}^{2} \bigl(2F_{k-1}F_{k+1}-F_{k}^{2}\bigr)\bigr\} \end{aligned}$$

is a $D(L_{k}^{2})$-quadruple.

Dujella and Ramasamy [32] considered the Fibonacci numbers and $D(4)$-quadruple. They proved that the set

$$\begin{aligned} \{F_{2k},5F_{2k},4F_{2k+2},4L_{2k}F_{4k+2} \} \end{aligned}$$

is a $D(4)$-quadruple. Also, they considered integer solutions of the Pell equations by using a $D(4)$-quadruple.

In the future work, we are planing to study $D(n)$-quadruples for the sequences mentioned for some *n*.

## Conclusion

In this work, we deduced some new results on Pell, Pell-Lucas, and balancing numbers including sums, divisibility properties, perfect squares, and integer solutions of some specific Pell equations.
